# Real-time tablet-based resuscitation documentation by the team leader: evaluating documentation quality and clinical performance

**DOI:** 10.1186/s13049-016-0242-3

**Published:** 2016-04-16

**Authors:** T. Grundgeiger, M. Albert, D. Reinhardt, O. Happel, A. Steinisch, T. Wurmb

**Affiliations:** Institute Human-Computer-Media, Julius-Maximilians-Universität Würzburg, Oswald-Külpe-Weg 82, 97074 Würzburg, Germany; Department of Anaesthesia and Critical Care/Section Emergency Medicine, University Hospital of Würzburg, Oberdürrbacher Strasse 6, 97080 Würzburg, Germany

**Keywords:** Cardiac arrest documentation, Cardiopulmonary resuscitation, Simulation, No-flow fraction

## Abstract

**Background:**

Precise and complete documentation of in-hospital cardiopulmonary resuscitations is important but data quality can be poor. In the present study, we investigated the effect of a tablet-based application for real-time resuscitation documentation used by the emergency team leader on documentation quality and clinical performance of the emergency team.

**Methods:**

Senior anaesthesiologists either used the tablet-based application during the simulated resuscitation for documentation and also used the application for the final documentation or conducted the full documentation at the end of the scenario using the local hospital information system. The latter procedure represents the current local documentation method. All scenarios were video recorded. To assess the documentation, we compared the precision of intervention delivery times, documentation completeness, and final documentation time. To assess clinical performance, we compared adherence to guidelines for defibrillation and adrenaline administration, the no-flow fraction, and the time to first defibrillation.

**Results:**

The results showed significant benefits for the tablet-based application compared to the hospital information system for precision of the intervention delivery times, the final documentation time, and the no-flow fraction. We observed no differences between the groups for documentation completeness, adherence to guidelines for defibrillation and adrenaline administration, and the time to first defibrillation.

**Discussion:**

In the presented study, we observed that a tablet-based application can improve documentation data quality. Furthermore, we demonstrated that a well-designed application can be used in real-time by a member of the emergency team with possible beneficial effects on clinical performance.

**Conclusion:**

The present evaluation confirms the advantage of tablet-based documentation tools and also shows that the application can be used by an active member of an emergency team without compromising clinical performance.

## Background

Precise and complete documentation of cardiopulmonary resuscitations is important for quality improvement [1], medicolegal reasons [2], and research [3]. For example, data such as the time to first defibrillation are used to predict survival rates of in-hospital cardiac arrest [4] and benchmarking and improving out-of-hospital cardiac arrests outcomes in Europe [5]. However, the validity of these results depends on the quality of the documented data. Precise documentation of intervention delivery times and complete documentation is difficult due to fast-paced intervention delivery, distorted time perception during the resuscitation [6], or errors when documenting from memory [7]. Research has shown that data quality of resuscitations can be poor [6–10].

Previous studies used information technology to improve the data quality of in-hospital cardiac arrests [7, 11–15]. All solutions but speech recognition [11], however, require a designated person for documentation. In Germany, an active member of the emergency team – usually a senior anesthesiologist – is responsible for documentation of in-hospital cardiac arrests. Furthermore, tablet-based [13, 16] or desktop-based solutions [12] have been evaluated by comparing tablet or desktop-based documentation vs. paper-based documentation when watching video recordings or live simulations of simulated resuscitations. In all studies, the documenting person was not involved in the actual resuscitation. To our knowledge, the effect of the documentation task on clinical performance has not been considered.

In the present study, we evaluated an existing, tablet-based application [17] for real-time documentation of in-hospital resuscitations by a member of the emergency team. To this end, anesthesiologists participated in a simulated resuscitation and had to document the resuscitation treatment using the tablet-based application (“the Application”) or the hospital information system (“the HIS system”). The latter procedure represents the current documentation method and is, therefore, a good benchmark to test the Application.

We expected more precise intervention delivery times, a more complete documentation, and a faster final documentation at the end of the resuscitation when using the Application compared to using the HIS system. Considering clinical performance, we assessed adherence to guidelines for defibrillation and adrenaline (epinephrine) administration based on European Resuscitation Council guidelines [18], no-flow fraction, and time to first defibrillation.

## Methods

### Setting

The medical emergency management of the University Hospital of Würzburg is based on a central medical emergency team. It consists of a senior anesthesiologist, one intensive care nurse, and a resident anesthesiologist. The senior anesthesiologist has special qualification in emergency medicine and intensive care medicine. The nurse has special qualification in intensive care medicine and special training in resuscitation. The team is equipped with a full scale defibrillator (M-Series, Fa. Zoll Medical Deutschland GmbH, Köln, Germany), Advanced Cardiac Live Support equipment, and difficult airway management equipment. The team is based in the intensive care unit of the Department of Anaesthesia and Critical Care and will be at any site within a maximum of 10 min after the emergency call. On scene, the team will cooperate with the medical staff serving as first responders. The members of the emergency team are scheduled for team training once a year. The training takes place in the simulation center of the department of anesthesiology.

### Study design

We used a between subject design with the groups Application vs. HIS system. The data was collected across 10 days in April and March 2015 in the simulation centre of the Department of Anaesthesia at the University Hospital of Würzburg. The manikin was the *Resusci Anne*® *Simulator* (Laerdal, Stavanger, Norway). The local university ethics committee approved the study. Written informed consent was obtained from all participants.

### Documentation systems

**Fig. 1:**
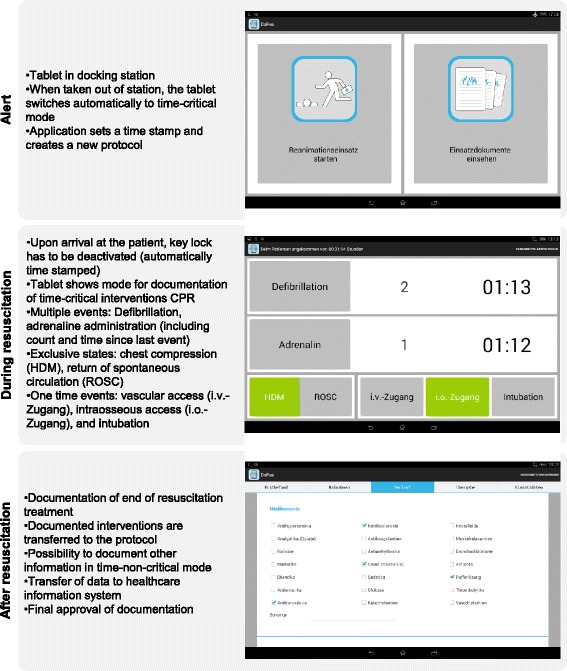
Summary of workflow for the Application when alerted, during the resuscitation, and after the resuscitation. For more information see Reinhardt et al. [17]

The Application supports documentation from alert until final documentation after the treatment has ended. In short, the Application has a time-critical and time-non-critical mode. The time-critical mode offers the opportunity for easy real-time documentation of interventions and associated intervention delivery times such as adrenalin administration or intubation. Because defibrillation and adrenaline administration occur multiple times during resuscitation, the Application counts the absolute number of interventions and indicates the total time elapsed after the last intervention. All data entered is stored in a protocol and can be accessed via the time-non-critical mode at a later point in time. In the time-non-critical mode, the anesthesiologists can edit the protocol and enter additional information such as patient’s consciousness or respiration during the emergency treatment (Fig. 1).

The HIS system is a SAP-based interface (SAP Deutschland SE + Co. KG, Walldorf, Germany). The emergency protocol consists of different pages which correspond to the different phases of an emergency (findings/medical history, interventions/resuscitation, clinical course, transfer/handing over, mission data). The content of the HIS system is based on the suggestions of the German Resuscitation Registry (http://www.reanimationsregister.de) [3]. The content of the Application was the same as in the HIS system.

### Procedure

**Fig. 2:**
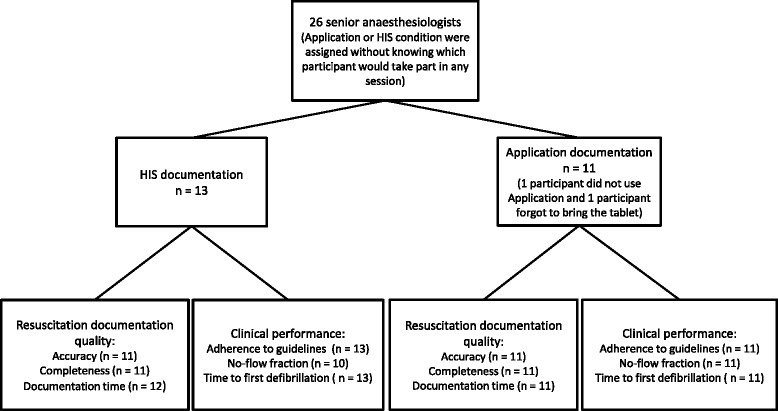
Flow chart of data collection. The *n* in the far right boxes indicate the number of participants for each analysed depended variable. Data are missing due to technical failures or immediate work obligations of participants. HIS: Hospital Information System

A total of 26 anaesthesiologists (all members of the local resuscitation staff) were recruited via email. In the email, we told participants that we plan to compare two different approaches for in-hospital resuscitations documentation. We did not give further details about the specific conditions. All participants were familiar with the HIS system and tablet computers in general. Without previous knowledge of the actual participants, we determined which session included the Application or the HIS system (Fig. 2). Anaesthesiologists participated depending on their work schedules.

We prepared two different scenarios (Tables 1 and 2; PE: acute pulmonary embolism with rapid deterioration, HK: consecutive cardiac arrest and acute cardiac arrest resulting from hyperkalemia). On each day, we conducted two sessions (one in the morning and one in the afternoon) and each session included one of each scenarios. The documentation method was counterbalanced in relation to time of day.

**Table 1:** Scenario: Acute pulmonary embolism with rapid deterioration and consecutive cardiac arrest

Background	Patient on ward, 3 days after liver-surgery, ASA 2, was moved from intensive care unit to ward yesterday. When the physiotherapist was trying to mobilize the patient for the first time, the patient collapsed and breathing started to get worse. At first, the patient was still responding and moaning about having difficulties to breath. Insufflation of oxygen over a mask was started and an emergency call was made.
Emergency call	“This is ward XY calling. Please come quickly. One of our patients is not breathing properly.”
Situation	On arrival of the emergency team the patient was lying in his bed, breathing fast and noisily, unresponsive, with arterial hypotension and tachycardia and arrhythmia. After handover to the emergency team the patient deteriorated rapidly. Breathing ceases and the heart rhythm quickly changed to pulseless ventricular tachycardia and further to ventricular fibrillation requiring cardiopulmonary resuscitation.
Cause for cardiac arrest	Acute pulmonary embolism

**Table 2:** Scenario: Acute cardiac arrest resulting from hyperkalemia

Background	Patient on ward, 1 day after surgical debridement of chronic wound, ASA 3, nephropathy requiring renal dialysis. Dialysis was paused yesterday because scheduling for surgery. Patient was found unresponsive, not breathing by ward staff. Emergency trolley was obtained and emergency call was made.
Emergency call	“This is ward XY calling. Please come quickly. One of our patients is unconscious and not breathing.”
Situation	On arrival of the emergency team the patient was lying on the floor besides his bed. He was unconscious, pulseless and not breathing. Cardiopulmonary resuscitation had been started. The paddles of the ward’s automated external defibrillator were already placed on the chest, but the device was still turned off and no defibrillation has been performed so far. The patient has not been ventilated. The heart rhythm was a ventricular fibrillation.
Cause for cardiac arrest	Hyperkalemia

In each session, six hospital employees (all members of the hospital’s resuscitation staff) participated. In each scenario, the emergency team leader was a senior anesthesiologist. The five further hospital employees participated in each session and were either part of the emergency team (two persons) or pretended to be other staff members or bystanders (three persons).

Participants in the Application group received a three-minute training to get familiar with the Application. Participants were instructed to document as many interventions as possible (start time of cardiopulmonary resuscitation, defibrillations, etc.) during the simulated resuscitation. After the training, the tablet was placed in a docking station located in a separate room.

Participants in the HIS system group already knew the HIS system and no training was necessary. As in current practice, participants had to memorize the intervention and associated times during the resuscitation and document them in the HIS system at the end of the resuscitation.

All participants and their team members waited in the separate room until a member of the simulation team called and informed the team about the (simulated) emergency. Subsequently, the specific scenario was performed and video recorded. All clocks (control room, scenario room, cameras, and tablet) were synchronized before each session.

After the simulated scenario, the participant either had to complete the documentation with the Application or to perform the whole documentation using the HIS system. Finally, participants answered a demographic questionnaire.

### Data analysis

To assess intervention delivery times and completeness, we considered the following six interventions: start of emergency, arrival at patient, start of cardiopulmonary resuscitation, 1st defibrillation, 1st adrenaline administration, and intubation. The true intervention time (gold standard) was extracted from the videos, and we calculated the absolute difference between the true time and the documented time for each event. To investigate the overall effect of documentation completeness, we calculated how many of the six interventions per participant were documented using percentages. Finally, to assess the time needed for the final documentation, we compared the time needed for the documentation at the end of the scenario.

To investigate adherence to guidelines for defibrillation and adrenaline administration, we extracted the time intervals between each defibrillation and adrenaline administration starting with the 1st defibrillation. These intervals were subtracted by two minutes for defibrillation or four minutes for adrenaline administration. Based on ERC-guidelines 2010 [18], the resulting absolute values were considered as deviations from the guidelines. The two minute interval between consecutive defibrillations included the time for rhythm check and defibrillator charging. This was consented by the team.

We defined no-flow time as the time of cardiac arrest in which no chest compressions were being performed. The no-flow fraction is the ratio between no-flow time and the total time of cardiac arrest. Finally, time to first defibrillation was defined as actual time of 1st defibrillation minus arrival at the patient (scenario HK) or onset of cardiac arrest (scenario PE). For no-flow fraction and time to first defibrillation calculations, we used the data recorded by the patient manikin (*SimPad*®, Laerdal, Stavanger, Norway).

For sample size calculation, we considered previous research using different documentation technologies [11, 12]. When expecting average differences of 30 s between the Application and the HIS system, an a-priori power analysis with 1–β = .95, α = .05, and a large effect (d = 1.5) resulted in a required sample size of 2 × 13 participants. All data were analysed using independent t-tests (means, M; standard deviation, SD) or Mann–Whitney U tests (median, Mdn; 25-percentile, quartile 1; 75-percentile, quartile 3) if the dependent variable was not normally distributed based on a Kolmogorov-Smirnov test. Statistical analysis was conducted using IBM SPSS Statistics for Windows, Version 21.0 (Armonk, NY: IBM Corp).

## Results

The demographics and scenario related measures of the final sample (Application: *n* = 11, HIS: *n* = 13) are summarized in Table 3. All variables showed no significant differences. Therefore, we did not observe any biases regarding, for example, the work experience of the participants. The final sample sizes of the two groups and the final number of outcome measures are shown in Fig. 2

**Table 3:** Demographic data, experience, and scenario length separated for Application and HIS system groups

Variable	Application (*N* = 11)	HIS system (*N* = 13)	Test statistic
Female/male gender	3/8	3/10	-
Age (years)^a^	38 (34;39)	37 (33;39)	U = 44.5, *P* = 0.300
Work experience as anaesthetist (years)^a^	9 (7;11)	7 (5;10,5)	U = 38.0, *P* = 0.151
Scenario length (mm:ss)	16:32 (15:32;17:30)	16:35 (16:02;17:38)	U = 81.5, *P* = 0.569

Data are presented as frequencies or median (25-percentile; 75-percentile)

^a^Data of two participants in the HIS system group are missing due to immediate work obligations at the end of the session

### Resuscitation documentation quality

As expected, the average deviation across all six interventions between actual intervention delivery times and recorded times was smaller in the Application group (Mdn = 0:38 min, quartile 1 = 00:28, quartile 3 = 01:42) compared to the HIS system group (Mdn = 2:51 min, quartile 1 = 01:30, quartile 3 = 03:58, U = 104.0, *P* = 0.003). The medians are shown in Table 4. Considering documentation completeness, there was no significant difference between the Application group (Mdn = 83 %, quartile 1 = 67, quartile 3 = 100) and the HIS system group (Mdn = 83 %, quartile 1 = 83, quartile 3 = 100, U = 76.5, P = 0.300).

**Table 4:** Differences in seconds between true time of an event and documented time depending on group (Application vs. HIS)

	Application group	HIS group
Start of emergency	27 (9, 31)	161 (51, 275)
Arrival at the patient	5 (3, 10)	102 (43, 181)
Start CPR	82 (16, 57)	159 (24, 317)
First defibrillation	49 (21, 69)	76 (33, 467)
First adrenalin administration	8 (4, 25)	223 (112, 350)
Intubation	107 (65, 237)	301 (136, 521)
Overall deviations	38 (28, 102)	171 (90, 238)

Values indicate median (25-percentile, 75- percentile)

*HIS* Hospital information system, *CPR* cardio pulmonary resuscitation

The analysis showed a significantly shorter final documentation time for the Application group (M = 8:04 min, SD = 3:49) compared to the HIS system group (M = 12:19 min, SD = 2:58, t(19) = −3.003, *P* = 0.007).

### Clinical performance

For the adherence to guidelines for defibrillation and adrenaline administration, the analysis showed no significant differences for the periods between each defibrillation (Application: Mdn = 0:51 min, quartile 1 = 00:35, quartile 3 = 01:10; HIS system: Mdn = 0:53 min, quartile 1 = 00:28, quartile 3 = 01:36, U = 71.0, *P* = 1.00) and each adrenaline administration (Application: M = 1:48 min, SD = 0:32; HIS system: M = 1:39 min, SD = 0:51; t(22) = 0.496, *P* = 0.63). For both groups, about 30 % of the defibrillations were conducted too early and about 55 % of the adrenaline was administrated too early with respect to current guidelines [18].

The no-flow fraction was significantly shorter in the Application group (M = 16.91 %, SD = 5.67) compared to the HIS system group (M = 22.44 %, SD = 6.10, t(19) = −2.151, *P* = 0.046).

The analysis of the time to first defibrillation showed no differences between the Application (Mdn = 2:05 min, quartile 1 = 01:29, quartile 3 = 04:27) and the HIS system group (Mdn = 2:37 min, quartile 1 = 01:33, quartile 3 = 03:54, U = 77.5, *P* = 0.73).

## Discussion

In line with previous research [13, 16], the Application improved precision of intervention time documentation by 78 % compared to the current local standard (HIS system). In the present study, the overall time of the resuscitation was rather short and the documentation was done immediately after the resuscitation. In the actual clinical context, the time is likely to be longer and documentation may be done much later. We therefore expect an even larger benefit of the Application in actual clinical settings. Furthermore, final documentation time was reduced by 34 %. This may be due to several reasons. First, the data entered during the resuscitation is automatically entered in the protocol. Second, the touch interaction may have made it easier for participants to navigate and complete the protocol. In an actual clinical setting, the tablet also enables the team leader to complete the protocol at any place. However, using the tablet requires the team leader to electronically transfer the data to the HIS system after finishing the documentation. This final step was not part of the scenario and would require further time. Finally, completeness of documentation was not improved. This may be due to the form-like structure of the HIS system that may have prompted participants to enter data.

Considering clinical performance, the data showed no difference between the current documentation method and the Application in relation to deviations from recommended defibrillation and adrenaline administration rhythms and time to first defibrillation. Considering the no-flow fraction, the value for the HIS system (22.44 %) was similar or even better compared to data from actual resuscitations by emergency teams (24 %) [19], ward nurses using a Automated External Defibrillator (40 %) [20], and simulated resuscitations (25 %) [21]. More interesting, using the Application resulted in a reduction of the no-flow fraction of 5.53 to 16.91 %. This is particularly important because studies have shown that even short interruptions of chest compressions affect survival rates [22–24]. The difference in no-flow fraction may be due to better coordination by the team leader when using the Application. In this case, the Application may have supported the team leader by serving as a cognitive aid. Such cognitive aids [25], for example, have been developed for identifying reversible causes of a cardiac arrest [26] or assisting in resuscitations [27]. However, if the Application served as cognitive aid, one may also have expected improved defibrillation and adrenaline administration rhythms closer to the guidelines. Overall, the results show that using the Application for real-time documentation did not result in worse clinical performance compared to not using the Application. The Application does not seem to distract the senior anaesthesiologist during the resuscitation. However, we do not have data on whether the Application distracted the team leader from other tasks such as identifying the reversible causes of cardiac arrest. This was not part of the study protocol. Further studies are needed to get more valid information about the risks and benefits while using the Application during cardiopulmonary resuscitations.

In the present study, one potential source of bias might have been the two distinct scenarios. However, when split by scenarios, the data showed the same result pattern as the reported results. Furthermore, as reported previously [11], manual records are only recorded to the nearest minute whereas tablet-based records are recorded in minutes and seconds. However, when rounding the Application timestamped data to the nearest minute we still observed more precise intervention delivery times in the Application group compared to the HIS group. Finally, a full-random assignment would have been preferable but was not possible. Therefore, we assigned the respective conditions (Application vs. HIS) without knowing which participants would take part in any session. Furthermore, all collected control variables indicate no differences between groups (Table 3).

## Conclusion

The present evaluation confirms the advantage of tablet-based documentation tools and also shows that the application can be used by an active member of an emergency team without compromising clinical performance. Research is needed to investigate whether specifically designed tablet-based documentation tools might even further improve clinical performance by supporting coordination of tasks and supporting a guideline conform resuscitations. Furthermore, we are currently implementing the Application and plan to investigate whether the observed benefits translate into practice.
